# The commissioning and validation of Monaco treatment planning system on an Elekta VersaHD linear accelerator

**DOI:** 10.1002/acm2.12507

**Published:** 2018-12-07

**Authors:** Jeffrey E. Snyder, Daniel E. Hyer, Ryan T. Flynn, Amanda Boczkowski, Dongxu Wang

**Affiliations:** ^1^ Department of Radiation Oncology University of Iowa Hospitals and Clinics Iowa City IA USA

**Keywords:** collapsed cone, heterogeneity, Monaco, Monte Carlo, SBRT, VMAT

## Abstract

Accurate beam modeling is essential to help ensure overall accuracy in the radiotherapy process. This study describes our experience with beam model validation of a Monaco treatment planning system on a Versa HD linear accelerator. Data were collected such that Monaco beam models could be generated using three algorithms: collapsed cone (CC) and photon Monte Carlo (MC) for photon beams, and electron Monte Carlo (eMC) for electron beams. Validations are performed on measured percent depth doses (PDDs) and profiles, for open‐field point‐doses in homogenous and heterogeneous media, and for obliquely incident electron beams. Gamma analysis is used to assess the agreement between calculation and measurement for intensity modulated radiation therapy (IMRT) and volumetric modulated arc therapy (VMAT) plans, including volumetric modulated arc therapy for stereotactic body radiation therapy (VMAT SBRT). For all relevant conditions, gamma index values below 1 are obtained when comparing Monaco calculated PDDs and profiles with measured data. Point‐doses in a water medium are found to be within 2% agreement of commissioning data in 99.5% and 98.6% of the points computed by MC and CC, respectively. All point‐dose calculations for the eMC algorithm in water are within 4% agreement of measurement, and 92% of measurements are within 3%. In heterogeneous media of air and cortical bone, both CC and MC yielded better than 3% agreement with ion chamber measurements. eMC yielded 3% agreement to measurement downstream of air with oblique beams of up to 27°, 5% agreement distal to bone, and within 4% agreement at extended source to surface distance (SSD) for all electron energies except 6 MeV. The 6‐MeV point of measurement is on a steep dose gradient which may impact the magnitude of discrepancy measured. The average gamma passing rate for IMRT/VMAT plans is 96.9% (±2.1%) and 98.0% (±1.9%) for VMAT SBRT when evaluated using 3%/2 mm criteria. Monaco beam models for the Versa HD linac were successfully commissioned for clinical use.

## 1 INTRODUCTION

Accurate beam modeling plays an important role in the overall accuracy of the radiation therapy treatment process. The International Commission on Radiation Units and Measurements specifies a total dose uncertainty tolerance of 5% in patients,^1^ and decreasing dose calculation uncertainty is a means of achieving this goal. Furthermore, the choice of dose calculation algorithm has been shown to have a clinically significant impact on local tumor control rates. For example, in non‐small cell lung cancer patients treated with stereotactic ablative radiation therapy a local control benefit was shown for patients whose treatment plan was generated using collapsed cone convolution vs a pencil beam algorithm.^2^ This illustrates the importance in accurately commissioning and validating radiotherapy beam models used in the clinic.

Monte Carlo (MC) algorithms are the gold standard for dose computation in radiation therapy. MC dose engines simulate particles, track individual interactions and secondary generated particles, and tally dose deposition in a medium. Interactions at any given simulation step are determined through random number generation, the cross section of the respective stochastic process, and particles or photons are transported until their energy falls below a user‐specified cutoff energy.^3^ Due to the stochastic nature of these calculations, the calculated dose is subject to statistical uncertainty. In general, relative statistical uncertainty is proportional to the inverse square root of the number of histories generated. Large numbers of histories yield calculations with less statistical uncertainty but at the expense of increased calculation time.^4^

The continual progression of computing power has enabled MC calculation times on the order of several minutes, which is clinically acceptable. Multiple manufacturers including Elekta, Varian, RaySearch, and Accuray now offer treatment planning systems (TPSs) with MC dose algorithms. While many groups^5^, ^6^, ^7^, ^8^, ^9^, ^10^, ^11^, ^12^ have published their experience in commissioning MC‐based treatment planning models and algorithms, few references on Elekta's Monaco TPS are available.^10^, ^12^ Narayanasamy et al.^10^ evaluated the Monaco TPS's MC algorithms in low‐density heterogeneities and investigated the accuracy of volumetric modulated arc therapy (VMAT) dose distributions. Valdenaire et al.^12^ focused on the modeling of flattening filter‐free (FFF) beams in Monaco simulations and evaluating the accuracy of IMRT treatments and 3D conformal stereotactic body radiation therapy (SBRT) plans. Our study builds on this previous work by examining the Monaco TPS's calculation accuracy under several conditions which have not previously been reported. These include high‐density heterogeneities, obliquely incident electron beams, and VMAT SBRT treatments. For the current work, heterogeneity measurements are performed near tissue interfaces where electronic equilibrium is not present. Dose computations for photon Monte Carlo, electron Monte Carlo, and photon collapsed cone beam models are investigated for an Elekta Versa HD linear accelerator (Elekta AB, Stockholm, Sweden).

## 2 MATERIALS AND METHODS

The Monaco TPS (version 5.19.03d) was used for all calculations in this study. The Elekta Versa HD linear accelerator investigated in this work delivers photon energies of 6 MV (with flattening filter), 6 FFF (6 MV flattening filter free), 10 MV (with flattening filter), 10 FFF (10 MV flattening filter free), and 18 MV, and electron energies of 6, 9, 12, and 15 MeV. MC models were generated for each energy and modality. Collapsed cone convolution‐superposition (CC) models were also created for 6, 10, and 18 MV photon beams, with and without wedges. The CC models simulate the effects of physical motorized wedge, whereas MC models cannot be used for wedged fields in the Monaco TPS.

### 2.A Beam data collection and open‐field dosimetric verification

All beam scanning was conducted following the guidelines of TG‐106.^13^ A PTW MP3‐M water tank (PTW, Freiburg, Germany) and PTW's MEPHYSTO mc^2^ Navigation Software were used for all scanning and post processing of data. A PTW 31010 chamber with an active volume of 0.125 cm^3^ was used for all electron scanning and for profile scanning of photon field sizes of 20 × 20 cm^2^ and larger, along with percent depth dose (PDD) scanning of field sizes of 10 × 10 cm^2^ and larger. A Sun Nuclear Edge diode detector (Sun Nuclear Corporation, Melbourne, FL) was used for profile scanning of field sizes of 15 × 15 cm^2^ and smaller. A PTW 31014 ion chamber (0.015 cm^3^ active volume) was used for scanning of PDDs with field sizes of 7 × 7 cm^2^ and smaller, and for scanning of all wedged fields. Data collection for photon MC included open‐field profile scanning, PDD scans, output factor measurements, collimator scatter factors, and absolute dose measurement all performed at 90 cm source to surface distance (SSD). Photon CC beam models required additional scanning of wedged fields, diagonal scans, and wedge transmission factor measurements. Output factors for field sizes smaller than 5 × 5 cm^2^ were measured with the Edge diode detector and daisy‐chained to an ion chamber measurement, while ion chamber measurements alone were used for larger field sizes. Electron MC data included profiles in air at 90 and 70 cm SSD, profiles in water at 100 cm SSD, PDD measurements with and without applicators, output factors measured in air without the applicators, and absolute dose measurements. Collimator scatter factors were measured using the formalism provide in AAPM TG‐74^14^ using acrylic and brass mini‐phantoms.

In addition to open field data, the manufacturer provides users a set of eight “Express QA” plans. These fields have been described in detail by Narayanasamy et al.^10^ and are comprised of open‐field plans and a series of step and shoot IMRT plans. These plans are intended for verifying dose output and for fine tuning parameters such as MLC leaf tip offset position, MLC transmission, and leaf tip leakage. These fields were measured using a Sun Nuclear ArcCheck and sent to Elekta for final beam model generation.

#### 2.A.1 Profile validation

A CT scan of air was acquired and imported into the Monaco TPS. A 50 × 50 × 50 cm^3^ cube was contoured in air with an assigned electron density (ED) of 1.0 for the MC computations, and was set to be treated as water for the CC models (for CC models 1.0 ED is not pure water). Open fields were computed using a 2 × 2 × 2 mm^3^ dose grid and a statistical uncertainty of 1.0% per calculation for MC. Open‐field dose planes calculated by the Monaco TPS were exported for comparison with scanning data collected during commissioning. ScanDoseMatch (http://www.qxrayconsulting.com/sdm/), an open source scanning data analysis tool, was used to perform gamma analyses between modeled and measured data.^5^, ^15^ Gamma analysis was performed using a 2% and 2 mm passing criteria (relative mode) for both photons and electrons.

#### 2.A.2 Point‐dose and output factor validation

Photon models were evaluated by comparing TPS‐calculated point‐doses on the central axis and off axis against hand calculations for rectangular, square, and asymmetrically shaped fields. Comparisons were performed at SSD = 90 cm and SSD = 100 cm. Calculated output factors at different field sizes were compared against measurements. These tests were performed for MC and CC beam models. The model for motorized wedge was tested in the CC models by comparing profile consistency and dose accuracy in a homogenous water phantom.

Electron models were evaluated by comparing TPS calculated output factors for various cone and cut out combinations against measured values. Dose calculations in a homogenous water phantom were compared to dose values obtained from hand calculations. Additionally, obliquity and extended SSD calculations were tested and compared to dose measurements obtained from a 0.125 cm^3^ ion chamber which was cross calibrated against a PTW 30013 ion chamber with a valid ADCL calibration.

#### 2.A.3 Inhomogeneity measurements

**Figure 1:**
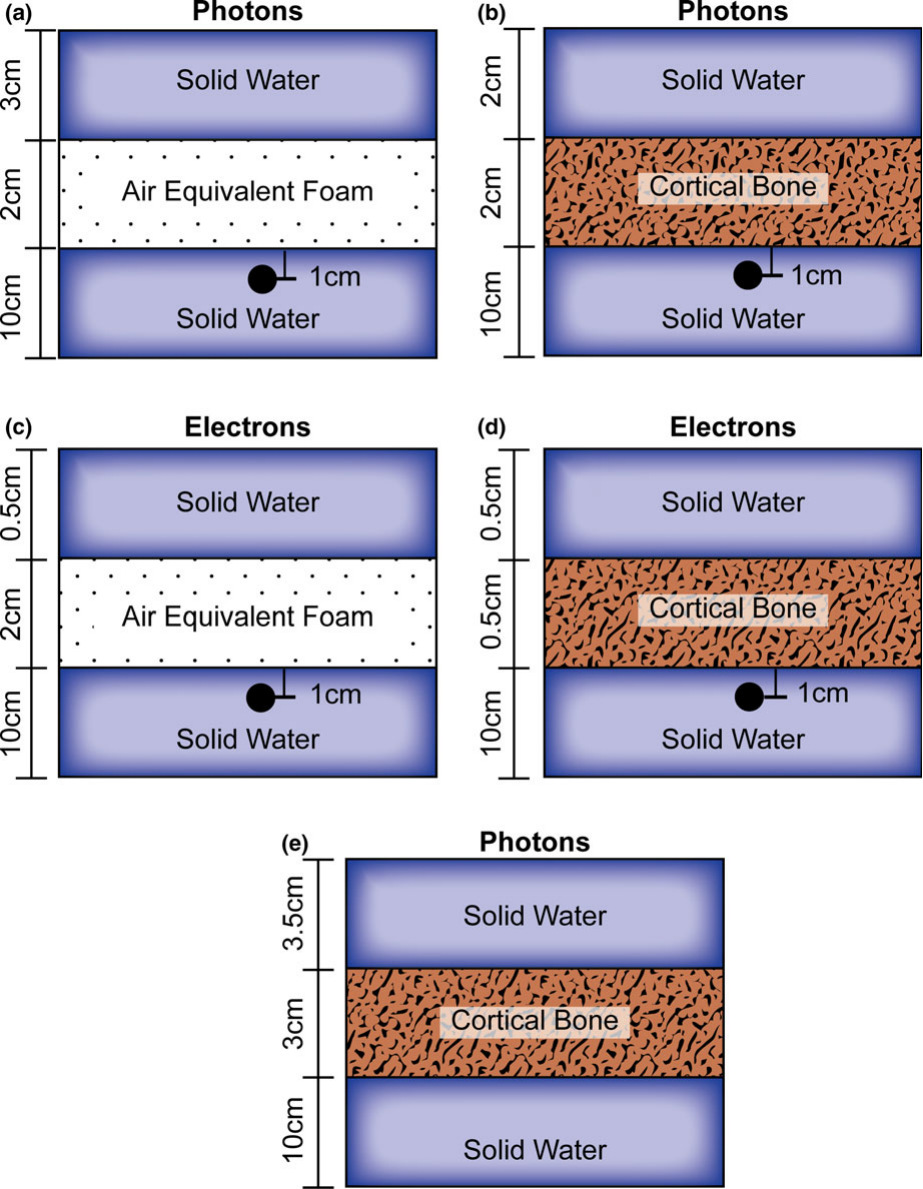
Heterogeneity slab geometry used for photon heterogeneity point‐dose measurements, (a) air, (b) cortical bone, electron heterogeneity point‐dose measurements, (c) air, (d) cortical bone, (e) and for 6 and 18 MV heterogenous PDD measurements with solid water and cortical bone

Inhomogeneity measurements were performed using solid water slabs with either air equivalent or cortical bone density equivalent slabs (CSP Medical, London, Ontario, Canada). Inhomogeneity measurements were performed for both photons and electrons using the same cross calibrated 0.125 cm^3^ ion chamber. Experimental setups are shown in Figs. 1(a)–1(d). CT scans of the setups were acquired and imported into the Monaco TPS. The drilled hole for the ion chamber insertion was contoured in the TPS and set to an ED of 1.0. This was done because the Monte Carlo calculation computes dose to medium and the cross‐calibrated ion chamber doses are related to that of the dose to water. When cortical bone density was used, high‐density streaking artifacts were contoured and set to solid water ED. The dose reference point in the TPS was placed such that it corresponded to the effective point of measurement within the ion chamber. Obliquely incident electron beams at angles of 10° to 27° were introduced with the air equivalent heterogeneity. 27° was chosen as the maximum angle because at 100 cm SSD this is the maximum angle that will allow clearance between a 10 × 10 cm^2^ electron cone and the external surface of the patient, as defined by modeling in Monaco.

In addition to point‐dose measurements, PDD measurements for 6 and 18 MV were performed in a heterogenous media consisting of solid water and cortical bone [Fig. 1(e)]. Measurements were conducted by placing pieces of Gafchromic EBT3 film (Ashland Advanced Materials, Bridgewater, NJ) at various depths along the central axis of a 10 × 10 cm^2^ field size beam at 100 cm SSD. Measurement points at the interfaces of the solid water and cortical bone are included as well as a measurement point inside of the cortical bone heterogeneity. The red channel was used for analysis and pixel values were converted to dose using a fitting equation from a Hurter and Driffield (H&D) curve along with ImageJ software. A CT scan of this same experimental setup was imported into Monaco where 6 and 18 MV plans were calculated and compared to measurement. All physical doses were normalized to the maximum depth dose and the results of this analysis are shown in Fig. 3.

### 2.B. Nondosimetric testing

A series of nondosimetric tests were performed in accordance with the recommendations AAPM TG‐53.^16^ Main components of this testing include: generation of CT number to ED curves, contour generation and 3D expansion accuracy, and creation of tolerance tables for use in Mosaiq oncology record and verify system (Elekta Inc. Atlanta, Georgia). Additionally, data export from Monaco to Mosaiq for multiple patient and phantom orientations was tested. CT to ED curves were created in Monaco from results of scanning an Electron Density CT Phantom (Gammex Inc., Middleton, WI) with known ED inserts using various CT kVp values. Additionally, the accuracy of the automatic rigid image registration was tested using datasets provided by AAPM TG‐132.^17^ These multimodality images include, CT, cone beam CT (CBCT), MRI, and PET. The images were imported with the known offsets provided by TG‐132 and the transformation matrix given in the TPS was compared to these known offsets.

### 2.C. Plan validation

Plan validations were performed using a Sun Nuclear ArcCheck. The ED of the ArcCheck was set following the recommendations specified by Sun Nuclear for use with Monaco. A virtual ArcCheck phantom was imported into the Monaco TPS with a manual ED override. The dose from a 10 × 10 cm^2^ field size at 100 cm source to axis distance (SAD) was calculated using the TPS and the entrance to exit diode ratio recorded. This setup is then delivered to the ArcCheck using the linear accelerator, and the measured entrance to exit diode ratio must match the calculated ratio to within 1%. Furthermore, the local gamma using 2% and 2 mm distance to agreement (DTA) passing criteria must be greater than 90%. If these criteria are not met, the ED of the virtual ArcCheck phantom must be iteratively adjusted.

#### 2.C.1 3D‐CRT plan validation

The collapsed cone model was validated by importing a CT dataset of a previously treated prostate cancer patient and generating 3D‐CRT plans. One, three‐field technique plan incorporating parallel opposed wedged fields and an AP beam, and one four‐field box technique plan was created for each CC modeled energy (6, 10, and 18 MV). All plans were delivered to the ArcCheck and evaluated using gamma analysis with absolute dose, global normalization, a low dose threshold of 10%, and passing criteria of 3% and 2 mm. A single point‐dose measurement was performed using a 0.125 cm^3^ ion chamber inserted into the ArcCheck. Point‐dose measurements were scaled by the ratio of mass energy‐absorption coefficient of water to that of the medium.^18^ This is required because the dose calculated in the virtual phantom is dose to poly(methylmethacrylate) (PMMA) while the dose measured by the ion chamber is related to a dose to water measurement. Failure to do this will lead to a systematic discrepancy in point‐dose measurements.

#### 2.C.2 IMRT/VMAT plan validation

IMRT and VMAT treatments were commissioned using select datasets provided by TG‐119^19^, ^20^, ^21^ as well as previously treated patient datasets from our institution. Test plans were selected to cover a range of treatment sites with corresponding energies that would likely be used clinically. The “C shape” CT dataset and structure set were imported into Monaco, and 1 IMRT and 1 VMAT plan were created for each energy (eight total plans on this dataset); target and OAR doses were designed to meet the “harder C shape” objectives. Head and neck and prostate datasets from TG‐119 and previously treated patients were also imported and planned with VMAT treatments, where 6 MV and 6 FFF energies were used for head and neck, and 10 MV and 10 FFF were used for prostate. All VMAT plans generated on TG‐119 data sets used two full arcs for VMAT plans or nine equally spaced beams for IMRT. The previously treated patient plans used two full 360° arcs. All of the TG‐119 datasets and the clinical head and neck and prostate plans were generated using 2 Gy dose per fraction schemes.

Additional VMAT SBRT plans were generated on previously treated patient datasets which included sites of the lung and pelvic lymph nodes. VMAT plans were generated using 6 MV and 6 FFF for lung sites, while 10 MV and 10 FFF energies were used for pelvic lymph node sites. The lung and pelvic lymph node sites were planned using an SBRT dose and fractionation scheme of 54 Gy in three fractions and 24 Gy in three fractions, respectively. High dose per fraction SBRT techniques create steep dose gradients in the target and immediate anatomical vicinity and are generally used for small targets which are deviations from standard plans and are therefore analyzed separately in Table IX. The pelvic lymph node plan was created using a single 360° arc, while the lung treatments used two partial arcs with angles chosen to avoid the contralateral lung. A single arc was chosen for the pelvic lymph node plans as this is our institutions current standard and furthermore the work of Kang et al. has found that there is no significant dosimetric difference between single arc and two arc VMAT plans for prostate SBRT treatments in terms of tumor control probability or normal tissue complication probability.^22^ QA plans were copied to the virtual ArcCheck phantom, calculated using a 2 × 2 × 2 mm^3^ dose grid. Gamma analysis was performed following the recommendations of TG‐218 where 3%/2 mm passing criteria was used with absolute dose, global normalization, and a low‐dose threshold of 10%.^23^

## 3 RESULTS

### 3.A Beam data collection and open‐field dosimetric verification

Beam data was collected such that MC models could be generated for all photon and electron energies and that additional CC models could be made for 6, 10, and 18 MV. The express QA field measurements were used by the vendor to determine parameters specific to the machine being modeled including MLC leaf tip offset position, MLC transmission, and leaf tip leakage. Proper determination of these physical machine parameters will optimize the performance of the MC dose calculation for advanced treatment planning techniques like VMAT and IMRT.

#### 3.A.1 Profile verification

**Figure 2:**
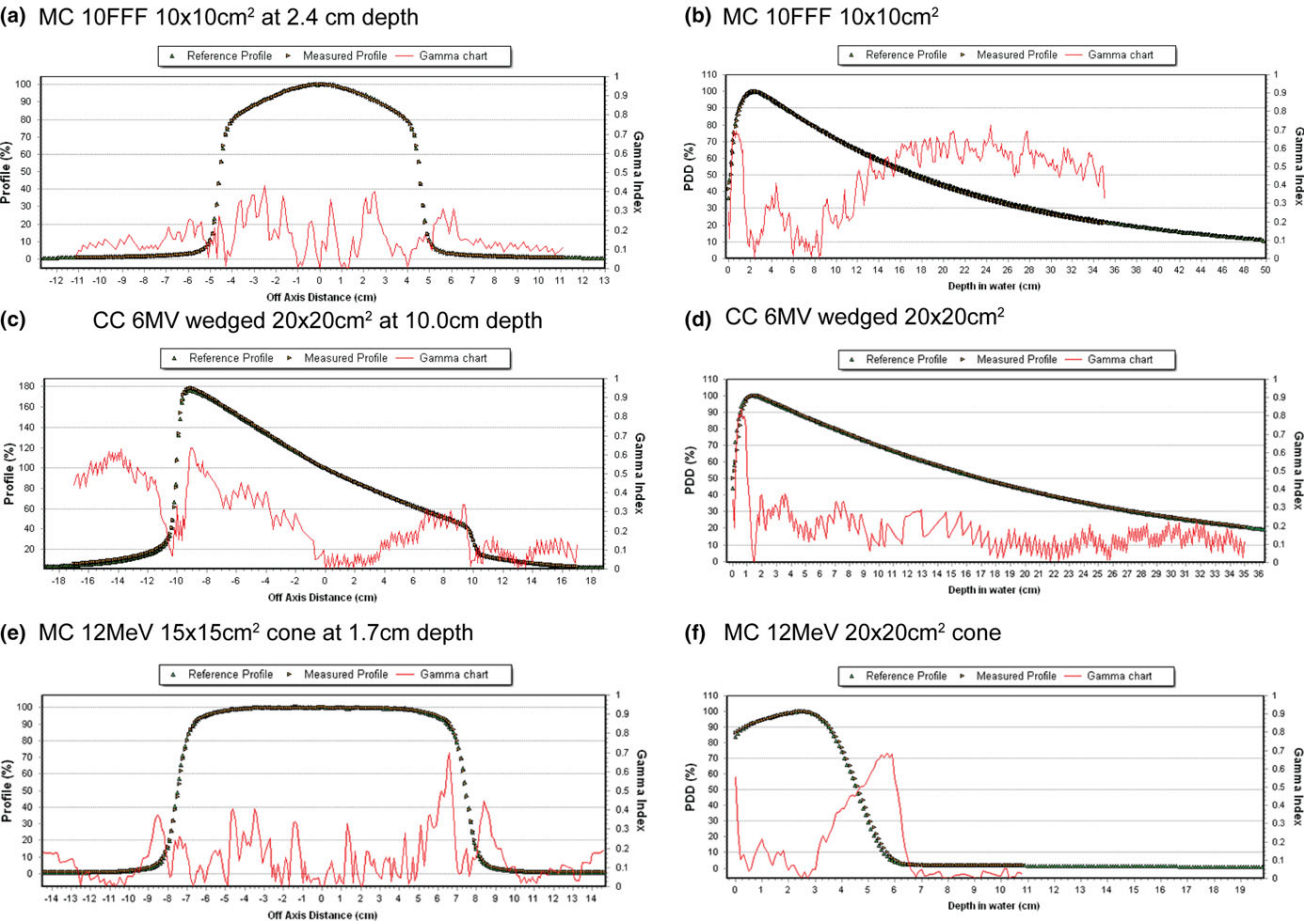
Gamma analysis between measured and calculated data for (a) Monte Carlo (MC) 10 Flattening filter free (FFF) 10 × 10 cm^2^ cross plane profile at 2.4 cm depth, (b) MC 10FFF 10 × 10 cm^2^ percent depth dose (PDD), (c) collapsed cone (CC) 6 MV wedged 20 × 20 cm^2^ in plane profile at 10.0 cm depth, (d) CC 6 MV 20 × 20 cm^2^ PDD, (e) Electron MC 12 MeV 15 × 15 cm^2^ cone in plane profile at 1.7 cm depth and, (f) MC 12 MeV 15 × 15 cm^2^ cone PDD

Sample results comparing modeled and measured profile data plotted with corresponding 2% and 2 mm gamma criteria are shown in Figs. 2(a)–2(f). Good agreement is observed for both MC and CC models, with all points passing the gamma analysis except in some circumstances where gamma values above 1.0 are observed in the tail region of profiles for field sizes of ≥30 × 30 cm^2^.

#### 3.A.2 Point‐dose and output factor validation

Open‐field dosimetric point‐dose validations were performed for both MC and CC models. Seventy‐nine (79) dose points were evaluated for each of the five photon energy MC models (395 points total) and 93 dose points were evaluated for each of the three photon energy CC models (279 points total). Across all data, 99.5% and 98.6% of TPS calculations are within the 2% tolerance recommended by Medical Physics Practice Guidelines 5a^24^ for the MC and CC algorithms, respectively. All calculated output factors in square fields at the depth of 10 cm for photon MC and CC models are within 2% of hand calculations and the majority is within 1%. Sample data points are shown in Table 1. Wedge field point‐dose validation was performed for all CC models and all of the calculated doses are within 2.5% of hand calculations (Table 2).

**Table 1:** Photon open‐field point‐dose verification

Model	Energy	SSD (cm)	Field size (cm²)	Depth (cm)	% (Meas‐Calc)/average
MC	6 MV	90	10 × 10	20.0	0.2
		100	40 × 5	1.5	−0.7
	6 FFF	90	2 × 2	5.0	0.6
		100	40 × 40	10.0	0.0
	10 MV	90	30 × 30	15.0	−0.5
		100	5 × 20	2.2	−1.6
	10 FFF	90	30 × 30	2.4	0.1
		100	5 × 5	5.0	−0.7
	18 MV	90	10 × 10	20.0	0.4
		100	5 × 40	3.0	−2.1
CC	6 MV	90	3 × 3	5.0	0.2
		100	20 × 20	1.5	−1.4
	10 MV	90	30 × 30	10.0	−0.9
		100	2 × 2	2.2	−2.5
	18 MV	90	5 × 5	10.0	0.7
		100	40 × 40	3.0	−0.4

**Table 2:** Wedged field point‐dose verification with CC algorithm

Energy	SSD (cm)	Field size (cm²)	Depth (cm)	% (Meas‐Calc)/average
6 MV	85	8 × 8	15	0.3
	90	5 × 5	5	−1
	110	20 × 10	5	−0.4
10 MV	80	20 × 10	25	−1.7
	90	10 × 10	15	0.6
	100	10 × 6	5	−1
18 MV	80	30 × 30	25	−2.2
	90	10 × 10	15	−0.3
	100	20 × 10	5	−2.4

Electron beam TPS point‐dose calculations in a homogenous water phantom yielded results that are within 4% of hand calculations at all evaluated points (Table 3); 92% (25 points evaluated) are within 3% of hand calculations evaluated across all electron energies. Output factors derived from TPS calculated dose values for field sizes greater than 3 × 3 cm^2^ agree to within 3% for all energies, cone, and cutout sizes tested. Calculated output factors for field sizes less than 3 × 3 cm^2^ show deviations of as much as 6.4% from measurement. Select results from output factor comparison are shown in Table 4. All point‐dose calculations at an extended SSD of 110 cm, shown as part of Table VI, are within 4% of measurement except for 6 MeV which is on a high‐dose gradient.

**Table 3:** Electron point‐dose verification

Energy	Cone (cm²)	Cutout (cm²)	Prescribed IDL (%)	%(Meas‐Calc)/average
6 MeV	6 × 6	5 × 5	80	−0.7
	14 × 14	10 × 10	85	0.3
	25 × 25	15 × 15	85	0.6
9 MeV	10 × 10	8 × 8	80	−0.9
	20 × 20	12 × 12	80	1.3
	20 × 20	10 × 10	85	−1.6
12 MeV	6 × 6	2 × 2	80	−3.2
	6 × 6	6 × 6	85	−0.3
	20 × 20	17 × 17	85	0.6
15 MeV	10 × 10	8 × 8	90	−2.8
	14 × 14	14 × 14	80	−0.2
	25 × 25	20 × 20	85	−0.6

**Table 4:** Electron output factor verification

Energy	Cone (cm²)	Cutout (cm²)	OF % (Meas‐Calc)/average
6 MeV	6 × 6	2 × 2	6.4
	10 × 10	8 × 8	−0.6
	20 × 20	17 × 17	−0.5
	25 × 25	15 × 15	0.3
9 MeV	6 × 6	3 × 3	4.5
	14 × 14	10 × 10	1.8
	20 × 20	12 × 12	−1.8
	25 × 25	20 × 20	−1.6
12 MeV	6 × 6	4 × 4	0.5
	10 × 10	6 × 6	1.0
	14 × 14	12 × 12	0.8
	20 × 20	20 × 20	0.2
15 MeV	10 × 10	3 × 3	0.7
	14 × 14	12 × 12	−1.9
	20 × 20	12 × 12	−1.5
	25 × 25	10 × 10	−1.4

#### 3.A.3 Inhomogeneity measurements

**Figure 3:**
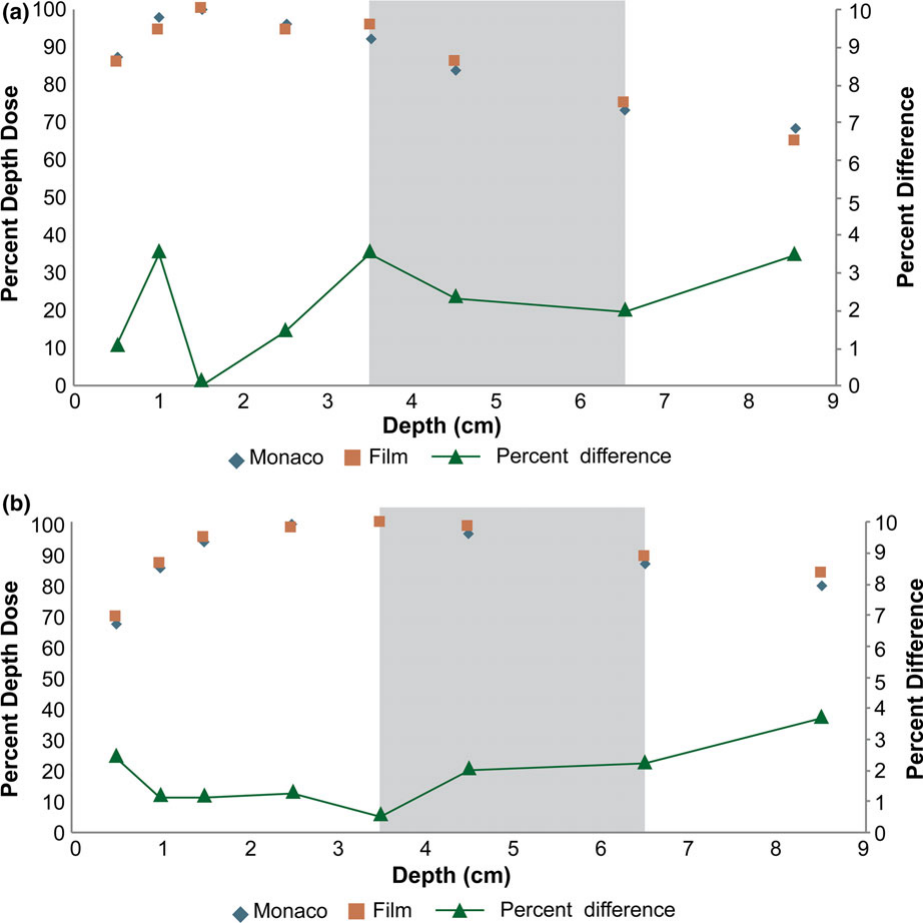
Heterogenous percent depth dose (PDD) Film Measurement vs Calculation (a) 6 MV and (b)18 MV PDD comparing Monaco calculation (blue) to EBT3 film (red) in a solid water and cortical bone (gray) medium, with the absolute magnitude percent difference plotted at each measurement depth (green)

Comparisons of TPS calculated and point‐dose measurements distal to the heterogeneities are shown in Table 5. All calculated point‐doses are within 2% of measurement for air equivalent heterogeneities and within 3% for cortical bone equivalent heterogeneities for both MC and CC photon beam models. The PDDs measured in heterogeneous media for 6 and 18 MV, shown in Fig. 3, are within 4% of calculation at all measurement points including tissue interfaces and within the cortical bone heterogeneity.

**Table 5:** Photon point‐dose heterogeneity measurements

Model	Energy	Gantry angle (°)	Heterogeneity	% (Meas‐Calc)/average
MC	6 MV	0	Air	−0.7
		20	Air	−0.2
		0	Bone	2.9
	6 FFF	0	Air	0.6
		20	Air	1.3
		0	Bone	−1.4
	10 MV	0	Air	0.4
		20	Air	0.6
		0	Bone	0.6
	10 FFF	0	Air	−0.2
		20	Air	0.9
		0	Bone	0.6
	18 MV	0	Air	1.3
		20	Air	2.0
		0	Bone	−0.9
CC	6 MV	0	Air	−0.9
		20	Air	−0.8
		0	Bone	1.2
	10 MV	0	Air	−1.2
		20	Air	−1.6
		0	Bone	−0.7
	18 MV	0	Air	−0.8
		20	Air	−0.9
		0	Bone	1.0

Electron point‐dose measurements in heterogenous media, shown in Table 6, for an air equivalent heterogeneity and enface beam are within 2%. The introduction of angle obliquity in addition to an air equivalent heterogeneity yields measurements which are within 3% of calculation for oblique angles of up to 27° for energies greater than 6 MeV. The 6‐MeV beam model agrees to within 1% of measurement for oblique angles of 20° or less, but has a −7.7% agreement between measurement and calculation at 27°. Point‐dose agreement between the TPS and measurement for electrons with a cortical bone density heterogeneity are within 5% for all energies except 6 MeV which was in a steep dose gradient. For 6 MeV, an exact match between the TPS and measurement is observed within 3 mm of the dose calculation point in the TPS.

**Table 6:** Electron point‐dose heterogeneity measurements

Energy	SSD (cm)	Gantry angle (°)	Heterogeneity	% (Meas‐Calc)/average
6 MeV	110	0	None	−10.5^a^
	100	0	Air	−0.4
	100	10	Air	0.0
	100	20	Air	0.8
	100	27	Air	−7.7
	100	0	Bone	−23.5^a^
9 MeV	110	0	None	3.3
	100	0	Air	−1.2
	100	10	Air	0.0
	100	20	Air	−1.3
	100	27	Air	0.8
	100	0	Bone	0.6
12 MeV	110	0	None	1.2
	100	0	Air	−2.5
	100	10	Air	0.0
	100	20	Air	−2.6
	100	27	Air	2.5
	100	0	Bone	4.9
15 MeV	110	0	None	0.9
	100	0	Air	−1.7
	100	10	Air	1.6
	100	20	Air	−1.8
	100	27	Air	1.4
	100	0	Bone	4.1

a Steep dose gradient. An exact point‐dose match between measurement and TPS found within 3 mm.

### 3.B Nondosimetric testing

All contour expansions and TPS volume calculations performed meet the guidelines recommended in TG‐53. CT to ED curves were generated in Monaco for various kVp values. Monaco HU values were compared to HU values of the displayed image on the CT scanner and satisfactory agreement was observed. Rigid image registration was performed and the registration error for all image modalities is within the stated TG‐132 tolerance, that the error in any cardinal direction must be less than one half of the voxel dimension. The average error in any single cardinal direction is 0.1 ± 1.4 mm. Connectivity between Monaco and Mosaiq was established such that end‐to‐end tests could be performed and no errors in data export were observed. No significant modifications to our current clinical workflows are needed to deploy Monaco in the clinic.

### 3.C Plan verification

An ED of 1.144 was determined to best match the material composition of the ArcCheck used at our institution. At this electron density, the local gamma passing rate for a 10 × 10 cm^2^ field using 2% and 2 mm criteria is 96.3% and the calculated ratio of entrance to exit diode doses differed from measured by only 0.59%. Both values are within the stated guidelines provided by Sun Nuclear.

#### 3.C.1 3D‐CRT plan validation

Comparisons between TPS calculated plans and ArcCheck measurements are shown in Table 7. Gamma analysis at 3% and 2 mm yielded passing rates of greater than 95% for all plans measured. Point‐dose agreement is within 2% for all plans.

**Table 7:** CC plan measurements

Energy	Plan	Gamma (3%/2 mm)	Point‐dose ratio
6 MV	4 field box	99.3	1.017
	3 field with wedges	95.8	1.019
10 MV	4 field box	96.7	1.013
	3 field with wedges	96.5	0.997
18 MV	4 field box	97.3	1.010
	3 field with wedges	95.6	0.994

#### 3.C.2 IMRT/VMAT plan validation

IMRT and VMAT plans generated on TG‐119 datasets were successfully created such that target coverage and OAR sparing was within the stated tolerances. All dosimetric objectives are either better than the mean values reported in TG‐119 or within 1 standard deviation of the mean for all machine energies and techniques evaluated. The average gamma passing rate at 3% and 2 mm criteria for all measured plans is 96.9% with a standard deviation of 2.1% (Table 8). Average gamma passing rates by energy are shown in Table 8. The average gamma passing rate for the VMAT SBRT plans (Table 9) is 98.0 ± 1.9%. All VMAT SBRT plans pass the tolerance stated in TG‐218. In total, 18 of the 19 plans evaluated pass the TG‐218 tolerance level with no plans exceeding the action level.

**Table 8:** IMRT/VMAT plan measurement results

Energy	Average gamma (3%/2 mm)
6 MV	97.3 ± 1.9
6 FFF	97.6 ± 1.8
10 MV	96.0 ± 3.2
10 FFF	96.6 ± 0.7

**Table 9:** VMAT SBRT plan measurements

Energy	Plan	3%/2 mm
6 MV	Left lung	95.9
6 FFF	Left lung	99.2
10 MV	Pelvic node	100.0
10 FFF	Pelvic node	97.0

## 4 DISCUSSION

Accurate dose calculation is a major component in the radiotherapy process which helps ensure that the delivered dose meets the physician's prescribed dose. The MC dose calculation algorithm is considered to be the gold standard and more accurately calculates dose in areas of electronic disequilibrium such as heterogeneity interfaces.^25^ Recent technological advances have reduced the calculation time required by MC algorithms and enabled the commercial development of TPS systems such as Monaco and RayStation for use in clinical practice. Despite the increase in computation speed for MC algorithms, CC algorithms are still faster and may provide value in helping increase efficiency in busy clinics when generating simple 3D‐CRT or palliative plans where the added accuracy of MC is not required. Additionally, the Monaco MC calculation algorithm does not support the physical motorized wedge which is commonly employed in many 3D‐CRT plans. Taking these factors into consideration it may be beneficial for clinics using Monaco TPS to have both CC models and MC models available. This work outlines the data collection requirements for each model and discusses pertinent commissioning tests as defined in the literature.^16^, ^21^, ^24^, ^25^ Major roles of the physicist in the commissioning of Monaco include accurate data collection, uploading data to the Elekta server, and beam model validation upon receipt of the models.

The comparison of photon beam profiles and single point‐doses distal to air heterogeneities have been the focus of previous work.^10^ As part of our commissioning process, similar measurements were performed, which yielded gamma index values below 1.0 under all relevant conditions and point‐dose agreement between calculation and measurement less than or equal to 2%. These results are in support of the previously reported findings. Our work, nevertheless, adds to the previous reported literature by examining point‐doses distal to high‐density heterogeneities. Our findings show that better than 3% agreement can be expected between calculation and measurement in the presence of high density heterogeneities. Furthermore, the film measurements in Fig. 3 show better than 4% agreement between calculation and measurement even at tissue interfaces where electronic equilibrium is not present. These results have not been previously reported for this TPS, and illustrate the calculation accuracy of the MC algorithm used in Monaco.

In many clinics, electron doses are calculated via hand calculations or with TPS that can have large systematic dosimetric errors when parameters such as heterogeneity, extended SSD, and beam obliquity are introduced.^26^, ^27^ Here, homogenous phantom point‐dose measurements are tested along with the incorporation of the above mentioned inhomogeneity. Results from Tables 3, 4, and 6 show that in a homogenous phantom all TPS calculated point‐doses are within 4% of monitor unit calculations. In general, the largest deviations are observed for small field sizes where measurement of output factors is difficult due to ion chamber partial volume averaging effects and lack of lateral scatter equilibrium which decreases the overall accuracy of the measurement. Point‐dose measurements made with a 10 × 10 cm^2^ cone and cutout at a depth of *d*_max_ for each respective electron energy agreed to within 1% of calculation, which supports previous findings in the literature.^10^ Overall, the beam profiles calculated in homogenous water agree well with measurement, where all gamma analyses values are below 1 using 2% and 2 mm passing criteria. A slight depth dependence is seen in Fig. 2(f) for 12 MeV and a 20 × 20 cm^2^ cone, but this still yields gamma values of below 0.7, and values below 0.5 up to approximately 4.6 cm which is beyond the clinically useful range in terms of prescribing. All calculated points for energies greater than 6 MeV are within 3% of measurement for air heterogeneities even when oblique angles from 10° to 27° are introduced. Our results show that in this energy range beam obliquity does not decrease the accuracy of the eMC dose computation. Furthermore, energies above 6 MeV show better than 4% agreement between calculation and measurement at an extended SSD of 110 cm and better than 5% distal to a cortical bone heterogeneity. In cases of extended SSD, beam obliquity, and presence of heterogeneities these MC calculations will be more accurate than hand calculations or calculations performed with conventional electron dose calculation algorithms.

The 6 MeV eMC beam model calculations had excellent agreement with measurement in a homogenous water medium and for obliquity measurements of 20° or less. At oblique angles of greater than 20° the agreement with measurement begins to degrade as evidenced by the −7.7% difference between measurement and calculation (Table 6.) Additionally, −10.5% and −23.5% differences are observed between calculation and measurement at extended SSD and with the incorporation of a cortical bone heterogeneity, respectively. In the extended SSD and cortical bone cases steep dose gradients were present and an exact point‐dose agreement with measurement was found within 3 mm of the point of measurement in the TPS. Therefore, setup uncertainties and partial volume effects of the ion chamber may contribute to the magnitude of discrepancy between measurement and calculation. It is possible that the 6 MeV eMC model, despite matching commissioning data acquired in water, is inadequate in computing dose in situations deviating from the commissioning condition. Due to the systematic discrepancy of these measurements, we are investigating possible solutions with the vendor to improve their model in these scenarios without compromising the accuracy of the homogenous water calculations. The above setups represent extreme circumstances which are not routinely clinically observed. For these reasons this model has been approved for clinical use. Clinical judgment and possible measurement verification may be required when prescribing at extended SSDs, with high‐density heterogeneities, or where large angles of obliquity are present.

Plan validations were performed separately for CC and MC calculation algorithms and are intended to cover a range of commonly treated sites. The algorithm chosen for plan creation is intended to mimic clinical use where 3D‐CRT plans are to be generated using CC and IMRT and VMAT plans are made with MC. Average gamma passing rates at 3% dose difference (DD) and 2 mm DTA are 96.9 ± 1.3 and 96.9 ± 2.1 for CC and MC, respectively. All VMAT SBRT plans evaluated have 95.9% or greater of their points passing the gamma analysis at 3% and 2 mm criteria which exceed the tolerance limit stated in TG‐218. In total 94.7% (18/19) of VMAT/IMRT plans measured are within the tolerance limit of TG‐218 and no plan measurements exceed the action limit. These results compare favorably to the work of Narayanasamy et al.^10^ who, using a Monaco TPS for dose calculation, reported an average gamma passing rate of 95.0% with 3% DD and 3 mm DTA, which are less stringent criteria than reported in this study. This is in good agreement with the 95% confidence limit expectation. All plans were successfully exported to Mosaiq and delivered without any significant deviations to our institutions current workflows.

## 5 CONCLUSION

Measured beam profiles are in strong agreement with TPS calculations with 2% DD and 2 mm DTA gamma criteria. Point‐doses in homogenous and heterogenous media are in strong agreement with the recommended tolerances of MPPG 5a. All plan measurements pass the gamma criteria recommended in TG‐218. CC, photon MC, and eMC algorithms have been successfully commissioned and are ready for clinical implementation.

## CONFLICT OF INTEREST

The authors declare no conflicts of interest pertaining to this work.
